# Urogenital Epithelial Cells as Simple Markers of Estrogen Response in Infants: Methods and Applications

**DOI:** 10.1371/journal.pone.0077061

**Published:** 2013-10-16

**Authors:** Margaret A. Adgent, Gordon P. Flake, David M. Umbach, Virginia A. Stallings, Judy C. Bernbaum, Walter J. Rogan

**Affiliations:** 1 Epidemiology Branch, National Institute of Environmental Health Sciences (NIEHS), NIH, Research Triangle Park, North Carolina, United States of America; 2 Cellular and Molecular Pathology Branch, NIEHS, NIH, Research Triangle Park, North Carolina, United States of America; 3 Biostatistics Branch, NIEHS, NIH, Research Triangle Park, North Carolina, United States of America; 4 Children’s Hospital of Philadelphia and University of Pennsylvania, Philadelphia, Pennsylvania, United States of America; University of Miami School of Medicine, United States of America

## Abstract

Exposure to estrogen-mimicking chemicals during critical periods of development, such as infancy, may have adverse effects. However, these effects can be difficult to characterize in most epidemiologic studies. For example, growth of reproductive organs may be susceptible to estrogenic chemicals, but measuring it requires skilled ultrasound examination; timing of pubertal onset may be altered, but observing it requires long-term follow up. To address the need for a simple marker of response to estrogenic exposures in infants, we propose a novel application of a classic marker of estrogen response in adult women: cytological evaluation of urogenital epithelial cells. In this cross-sectional study of 34 female and 41 male infants, we demonstrate that epithelial cells can be obtained from swabs of the vaginal introitus (females) and urethral meatus (males), as well as from spun urine, and that these cells respond to differential estrogenic conditions, as indicated by the relative abundance of the superficial epithelial cell type. To model varying estrogen exposure, we sampled from infants who were either newborn (highly exposed to maternal estrogens), or 12 weeks old (12W) (negligibly exposed to estrogen). Newborns had a higher percentage of superficial cells (%S), as compared to 12W (mean ± standard error: 8.3 ± 1.8 vs. 0.9 ± 0.2) (p < 0.01), consistent with an estrogen response. This difference in %S from newborn to 12W was observed similarly for swab (-7.6 ± 1.7) and urine (-7.3 ± 2.6) specimens and for males (-9.6 ± 2.9) and females (-5.2 ± 2.1). Examination of urogenital epithelial cells can successfully demonstrate estrogen response in both sexes, using cell specimens collected from either swab or urine sampling. In future studies, this simple, non-invasive method may be applied to assess whether estrogen-mimicking chemicals produce an estrogenic response in infants.

## Introduction

The endocrine disruption hypothesis states that hormonally active pollutant chemicals are interfering with endocrine function in the general population.  Estrogen-mimicking compounds are perhaps the best studied of the endocrine disrupting chemicals. Exposure to these chemicals during critical periods of development, such as infancy, is of particular concern. However, while it is possible to measure an infant or child’s *exposure* to a potentially estrogenic compound, there are few feasible, well validated ways of assessing *response* [1].  For example, exposure to an estrogenic endocrine disruptor during infancy may alter the timing of pubertal onset, but such an outcome takes many years of follow up to observe. Previously, we have attempted to develop valid, reliable methods of assessing estrogen response in young infants. Our model for estrogen exposure exploits the large natural change that occurs between birth (high estrogen exposure) and later in infancy (low estrogen exposure).  We have shown that the uterus and male breast are large at birth, consistent with the known trophic effect of maternal estrogen, and that these organs shrink as the infant gets older and estrogen exposure is lost [2]. Accordingly, reproductive organ volume may be a useful measure for evaluating whether an infant is responding to a biologically relevant amount of a substance with estrogenic activity. These measures, however, are technically challenging to obtain--for example, evaluating the uterus requires ultrasound, and breast tissue can be difficult to distinguish from fat. 

The epithelial cells of the vagina and urethra are additional markers of estrogen response that are simple to collect and analyze. Papanicolaou [3] first observed hormone-responsive epithelial cells in adult women, correlating periodic cytological changes in the vagina with phases of the menstrual cycle. Responsive cells were subsequently identified in the distal urethra of both males and females [4–7], as both urethral and vaginal tissues have embryologic origins in the urogenital sinus [8]. The cells of the epithelium naturally progress, or “mature”, from basal/parabasal through intermediate to superficial types in response to hormonal exposures. Basal/parabasal cells predominate in low hormone life-stages (e.g., childhood, post-menopause), intermediate cells predominate under the influence of progesterone (e.g., the luteal phase of the menstrual cycle), and superficial cells, the most differentiated cell type, arise under the influence of estrogen [9]. Accordingly, a qualitative estimate of hormone exposure can be derived based on the proportion of each cell type in epithelial cell specimens collected from either vaginal smears or urinary sediment [10,11]. 

Urogenital epithelial cells in infants should exhibit responsiveness that is similar to that seen in adults. Superficial cells, for example, should be found in higher proportions in the neonatal period, due to high levels of exposure to maternal estrogen at term, than they are later in infancy, after the infant is no longer exposed to maternal estrogen, and endogenous estrogen production is low. Urethral epithelial cells collected from urine specimens [12,13] and vaginal cells from epithelial swabs [14,15] show this sequence. Here, we evaluate whether swabs from the distal urethra in infant males show the same pattern as cells from the vaginal introitus in infant females [15], and whether cells collected simultaneously from urine show the same pattern as those from swabs. We observe that urogenital epithelial cells, collected from swabs or urine specimens and from males or females, can serve as simple, minimally invasive, and readily interpretable markers of estrogenic response in early life, and should be useful in future studies of early life exposure to endocrine disrupting chemicals.

## Materials and Methods

### Ethics Statement

The National Institute of Environmental Health Sciences (NIEHS) Epidemiology Branch maintains a protocol for the collection and evaluation of specimens from healthy children. Studies on this protocol are usually small, involve minimal data collection, and produce study records that cannot be linked to the child once the study is complete. This overall protocol was cleared by the Institutional Review Board (IRB) at NIEHS, and specific uses of it are reported yearly. Subject recruitment for this study was carried out by the Division of Gastroenterology, Hepatology and Nutrition at the Children’s Hospital of Philadelphia (CHOP), and the specific study protocol was cleared by the CHOP IRB. A parent provided informed, written consent for the infant’s participation. 

### Study subjects

 Infants born between 37 and 41 weeks gestation, with a birth weight between 5 and 10 pounds, no known acute or chronic health condition, and no reported use of soy-based infant formula were eligible. All subjects were aged either ≤7 days (“newborn”) or 12 weeks (± 3 days) to represent high estrogen exposure and low estrogen exposure, respectively. This design is intended to correspond to the natural model of high maternal estrogen exposure near term birth, and low endogenous estrogen exposure later in infancy [16,17]. Thirty-four female and 41 male infants were recruited. In general, a swab- and urine-based collection was attempted for each participant; 9 subjects contributed only swab specimens; both a swab and a urine specimen were processed for 29 females and 37 males. 

### Swab cell collection

Epithelial cells were collected from the vaginal introitus in female subjects, or the meatus of the urethra in male subjects using a saline-moistened Polyester –tipped swab. The swab was rubbed on the vaginal introitus or urethral meatus for 5 seconds, and was then placed in a centrifuge tube with 10 ml of SurePath^TM^ (Becton Dickson and Company, Franklin Lakes, NJ) Preservative and stored at room temperature until processing. 

### Urine cell collection

 Urethral cells were collected from bagged urine samples obtained from both female and male infants (Hollister U-Bag Urine Specimen Collectors; 100 ml bag for newborns; 200 ml “pediatric” bag for infants aged 12 weeks). The urine bag was firmly adhered to clean perineal skin and remained on the infant for up to 1 hour, or until a 50 ml specimen was obtained. Once collected, specimens were drained from the bag into clean, sterile urine cups and placed on ice. For specimen volumes > 45 ml, the volume was split between two cups. A volume of SurePath^TM^ Preservative equal to the volume of the specimen was added to each cup. Specimens were thoroughly mixed and stored under refrigeration until processing.

### Specimen Processing

Tubes containing the vaginal or urethral swabs were agitated to ensure transfer of the collected specimen from the swab to the liquid. Both swab and urine specimens were then poured into centrifuge tubes and centrifuged for 10 minutes at 2000 rpm. The supernatants were decanted, and the remaining cell buttons mixed with SurePath^TM^ preservative by vortexing. The resuspended specimens were then processed by the standard GYN SurePath^TM^ processing procedure, and the slides stained with Sure Path PrepStain^TM^ (Becton Dickinson and Company, Franklin Lakes, NJ). Specimen processing was completed at TriCore Reference Laboratories (Albuquerque, NM).

### Maturation Indices

One pathologist (G.P.F., the “NIEHS pathologist”) evaluated all specimens with an Olympus BX51 light microscope, using the 40x objective to accurately assess the nuclear chromatin pattern. When possible, he counted 300 cells; if a specimen was hypocellular, he counted 100, or all the cells if a specimen contained only 50 - 99. Specimens with fewer than 50 cells or those that had inflammatory cells, bacterial or yeast overgrowth, or cytolysis were excluded. We also excluded 3 urine specimens with abundant small basal cells of probable urothelial origin. These cells, which come from the bladder, are not estrogen responsive. A large number of such cells would distort counts and possibly obscure an estrogenic effect. The NIEHS Pathologist was unaware of the age and sex of the child from whom the specimens were obtained and could not link a swab specimen to its paired urine pellet specimen; he was aware of the study design and its hypotheses. 

Swab and urine specimens were examined in separate batches, with all swab specimens evaluated first, and then all urine specimens. Each squamous cell in vaginal and urethral swab specimens was categorized into the following standard cytological types [9,18,19]:

• Basal/parabasal cells: relatively small cells with scant to moderate amounts of cytoplasm and large, round, centrally located nuclei containing vesicular chromatin.
• Intermediate cells: ranging in size from slightly larger than basal/parabasal cells to the size of superficial cells, with abundant, thin, transparent cytoplasm and nuclei containing fine vesicular chromatin.
• Superficial cells: similar to intermediate cells with abundant cytoplasm, but with small, pyknotic nuclei and no discernible chromatin pattern.

Cells in urine specimens, alternatively, were categorized only as “superficial” or “other,” since it was difficult to distinguish parabasal and intermediate epithelial cells of distal urethral origin from those of less responsive regions of the urogenital tract. 

A subset of specimens was also scored by an independent pathologist in the processing laboratory (TriCore Reference Laboratories, Albuquerque, NM). The processing laboratory was not blinded to the pairing of the swab and urine specimens and implemented less stringent inclusion criteria than the NIEHS pathologist. One hundred cells from each slide were counted and evaluated as described by Naib [20], using criteria similar to those described above. For specimens with fewer than 100 cells, 50 cells were counted and doubled. Specimens with fewer than 50 cells were not scored, but no other exclusion criteria were applied. 

### Statistical analysis

 Both swab and urine specimens were evaluated on the basis of the percent of superficial cells (%S), defined as the number of superficial cells divided by the total number of cells counted, similar to the “pyknotic index” used in previous studies [10]. For the data from the processing laboratory’s evaluation of the slides, the percentage of superficial cells was defined using a total cell count of 100 for all samples, regardless of whether 100 cells were counted, or 50 were counted and doubled. The contrast between high and low estrogen exposure was operationalized as the dichotomous contrast between newborn (high exposure) and 12-week old (low exposure) infants. To account for the pairing of collection methods within subjects, we used a generalized linear mixed model. We regarded the number of superficial cells in a specimen as having a binomial distribution with the number of trials given by the number of cells counted. The observed percent of superficial cells for each specimen is then an estimate of its binomial probability. The generalized linear model for mean percent of superficial cells used the identity link and included main effects for age (newborn, 12-week), sex (female, male), collection method (urine, swab) and all their two-way and three-way interaction terms. The variance model accounted for random variation in observed percent of superficial cells associated with subjects and with subject-by-collection-method interactions and specified separate variance parameters for each age group. The mean percent of superficial cells and standard errors for age groups, sexes, and collection methods correspond to the least squares mean values as estimated in the generalized-linear-mixed-model analysis. Sensitivity and specificity were calculated by treating “newborn” status as the gold-standard outcome. A score of ≥5% superficial cells was taken as the test’s informative cutpoint, on the basis of clinical observations reported by Collett-Solberg and Grumbach [21]. Analyses were completed using SAS 9.3 (SAS Institute Inc., Cary, NC). 

## Results

Of 141 processed specimens representing 75 infants, 94 specimens, representing 58 infants were successfully scored by the NIEHS Pathologist (66%, 36 complete swab-urine pairs) (Table 1). Most of the scored female specimens had enough cells present to obtain a total count of 300, whereas hypocellular specimens (50 - 100 cells) were common among scored males. Swabbed specimens were excluded due to insufficient quantity of cells (n = 11), inflammation (n = 7) and cytolysis (n = 1). Urine specimens were excluded due to inflammation (n = 15), insufficient quantity of cells (n = 8), excessive urothelial basal cells (n = 3) and bacterial overgrowth or cytolysis (n = 2). Specimen exclusions were most common among newborn males, where 80% of urine specimens and 46% of swab specimens were unevaluable, mostly on the basis of inflammation or insufficient quantity. Forty percent of 12-week female urine specimens were also excluded. The proportion of excluded specimens for other age, sex and collection method categories was < 25%.

**Table 1:** Characteristics of evaluated specimens (n= 141), n (%).

	Male				Female			
	Newborn		12-Week		Newborn		12-Week	
	Swab	Urine	Swab	Urine	Swab	Urine	Swab	Urine
Total Subjects	24		17		18		16	
Total Specimens	24	20	17	17	18	14	16	15
Scored Specimens								
Yes	13 (54)	4 (20)	15 (88)	13 (76)	15 (83)	12 (86)	13 (81)	9 (60)
No	11 (46)	16 (80)	2 (12)	4 (24)	3 (17)	2 (14)	3 (19)	6 (40)
Cells Counted								
300	3 (23)	1 (25)	7 (47)	3 (23)	15 (100)	12 (100)	12 (92)	8 (89)
100	10 (77)	2 (50)	7 (47)	9 (69)	0 (0)	0 (0)	1 (8)	1 (11)
50-99	0 (0)	1 (25)	1 (7)	1 (8)	0 (0)	0 (0)	0 (0)	0 (0)
Swab-Urine Pairs	20		17		14		15	
Scored Pairs	4		12		11		9	

**Figure 1:**
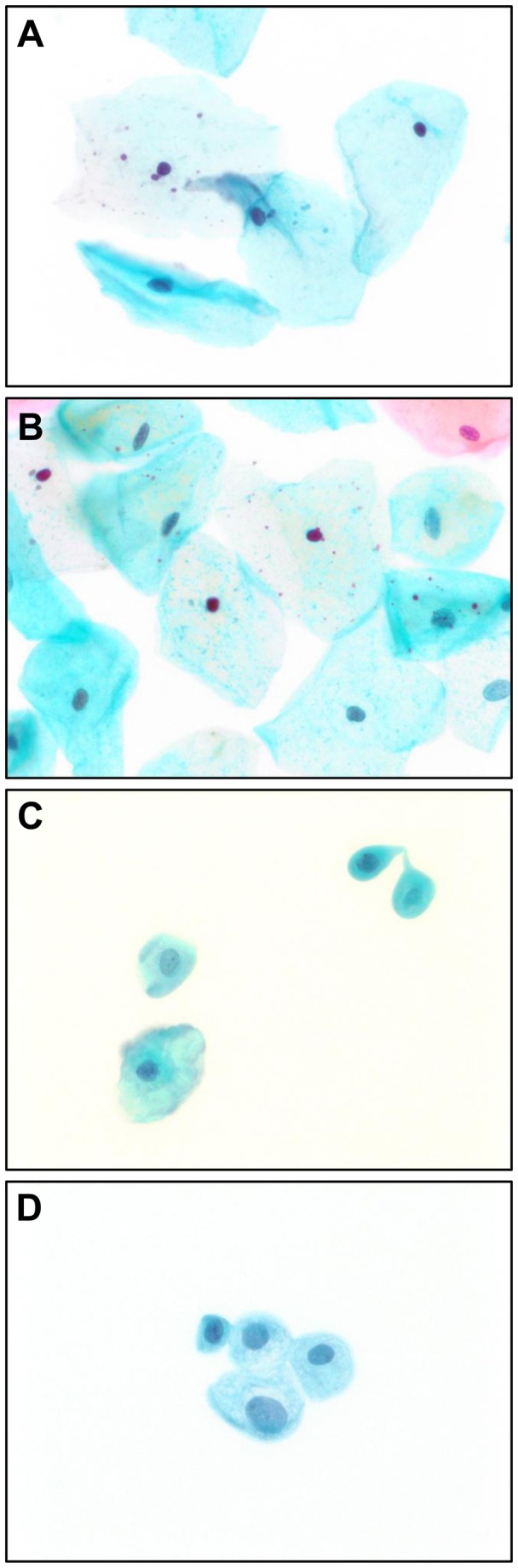
Newborn and 12 week urogenital cells collected from swab and urine specimens (female). A, Newborn Swab: Two superficial cells, characterized by small, dark, pyknotic nuclei and abundant cytoplasm in top right and left sides. Two intermediate cells, with slightly larger oval nuclei and more open chromatin patterns, are present in the middle and lower left. B, Newborn Urine: Two superficial cells, with small, pyknotic nuclei, are located in the center of the field. Most of the remaining cells are intermediates. C, 12-week Swab: Parabasal cells (upper right) and intermediate cells (lower left) are noted. Superficial cells were seen only rarely in this specimen. D, 12-week Urine: A cluster of four cells with parabasal to intermediate features. The cells show no features of superficial cells, which were completely lacking in this specimen.

Superficial and intermediate cells, indicated by large size and abundant cytoplasm, predominate in both swab and urine newborn samples (Figure 1A and 1B), with superficial cells being distinguished by the small, pyknotic nuclei. Alternatively, at 12 weeks, swab and urine specimens contained mostly the parabasal type, with some intermediate features (Figure 1C and 1D). 

**Figure 2:**
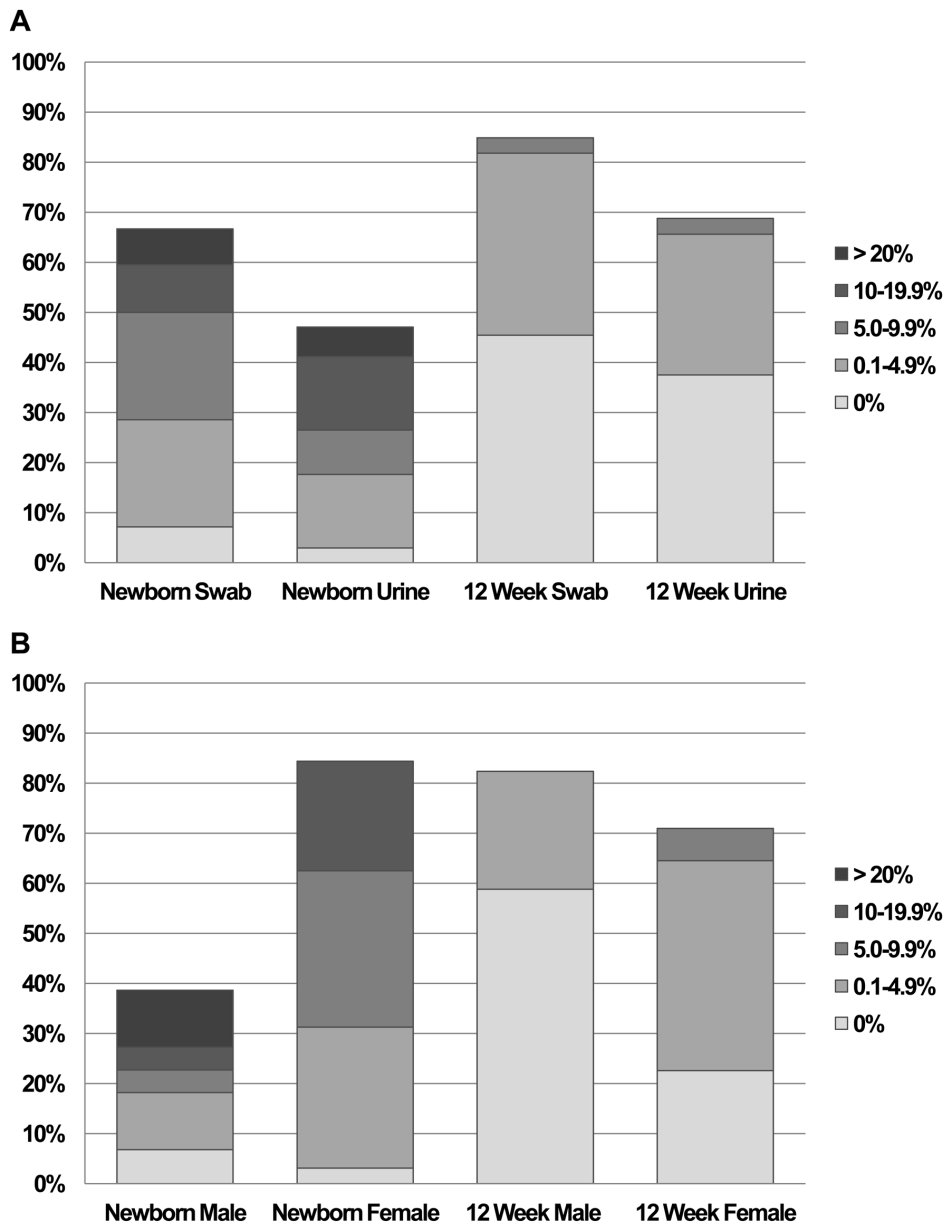
Distribution of percent superficial (%S) cells in newborn and 12-week specimens. Distribution of percent superficial (%S) cells in newborn and 12-week specimens according to **A**) cell collection method and **B**) sex. Color coding reflects the fraction of specimens within certain ranges of %S as indicated in the legend; overall height of vertical bars represents percent of specimens scored.

For newborn swab and urine specimens (Figure 2A), the percent of superficial cells was highly variable, ranging from 0 to >20%, with respective means and standard errors (SE) of 8.3 ± 1.7 and 8.4 ± 2.6. Alternatively, most 12-week specimens contained < 5.0% superficial cells (mean ± SE swab: 0.7 ± 0.3; urine: 1.2 ± 0.3). Similar patterns were observed when age groups were assessed by sex (Figure 2b). Using a cutpoint of ≥ 5% superficial cells, the sensitivity and specificity for this method were 59% and 96%, respectively. 

Our data analysis did not support any two- or three-way interactions between age, collection method, and sex (all p-values > 0.20). For example, the decrease in %S from newborn to 12 weeks was not statistically different for swab (-7.6 ± 1.7) and urine (-7.3 ± 2.6) specimens (p = 0.91) as well as for males (-9.6 ± 2.9) and females (-5.2 ± 2.1) (p = 0.22).  Consequently, the main effects alone summarize the important features of the data (Table 2). The mean percent of superficial cells differed significantly by age (p < 0.01; mean ± SE: 8.3 ± 1.8 and 0.9 ± 0.2, for newborn and 12-week specimens, respectively), but not by collection method (p = 0.84; mean ± SE: 4.5 ± 0.9 and 4.8 ± 1.3 for swab and urine specimens, respectively) or by sex (p = 0.57; mean ± SE: 4.1 ± 1.0 and 5.2 ± 1.4 for female and male specimens, respectively).

**Table 2:** The mean percent of superficial cells (%S) per specimen evaluated by the NIEHS Pathologist classified by infant age, sex and collection method.

Main Effects				Interaction Terms	
		**%S (95% CI)**	**p-value**		**p-value**
Infant Age	< 1 Week	8.3 (4.8, 11.9)	<0.001	Age ˣ Method	0.91
	12 Week	0.9 (0.4, 1.4)		Age ˣ Sex	0.24
Collection Method	Swab	4.5 (2.7, 6.3)	0.84	Method ˣ Sex	0.22
	Urine	4.8 (2.2, 7.4)		Age ˣ Method ˣ Sex	0.43
Infant Sex	Male	5.2 (2.2, 8.1)	0.57		
	Female	4.1 (1.9, 6.3)			

CI: Confidence Interval

Using specimen evaluations from the processing laboratory gave results that were broadly similar to those based on the NIEHS Pathologist’s readings. Among the 131 specimens evaluated, representing 70 subjects, 119 (91%) were scored; eight of the 12 excluded specimens (67%) were from newborn males. Like the NIEHS Pathologist’s findings, these laboratory evaluations showed a significant difference in the percent of superficial cells between newborn and 12-week specimens (p = 0.04; mean ± SE: 10.1 ± 1.7 and 5.4 ± 1.4, for newborn and 12-week specimens, respectively). Notably, these mean percentages tended to be higher than those generated by the NIEHS Pathologist, particularly for the 12-week specimens. Expectedly, results from the processing lab indicated no difference between sexes (p = 0.94; mean ± SE: 7.8 ± 1.6 and 7.6 ± 1.5 for female and male specimens, respectively) or between swab and urine collection methods (p = 0.19; mean ± SE: 8.2 ± 1.2 and 7.2 ± 1.2 for swab and urine specimens, respectively). However, uniquely among newborn males (n = 19 swabs; n = 15 urine), the mean percent of superficial cells was 3.6% greater (p < 0.01) for swab specimens than for urines. 

## Discussion

 In this study, we used the framework of the natural hormonal decline in early infancy [17] to demonstrate the utility of urogenital epithelial cells as simple, highly specific biomarkers of estrogenic response in infants. We observed that superficial cells markedly declined between the highly estrogenized state at birth and the low estrogen state later in infancy. We also observed that this process happens similarly between males and females. We failed to observe a difference between the superficial cells detected in specimens collected via swabbed epithelium or urinary sediment, suggesting that either method may be suitable. The cell processing described here is similar to that for vaginal cytology previously used to measure hormonal responses in adult women [22]. To our knowledge, this study is the first to demonstrate that epithelial cells can be successfully retrieved from a swab of the urethral meatus in young males. Both swab and urine collection methods provide a simple, interpretable measure of whether exposure to a biologically active amount of estrogen has occurred. The approach could be used throughout childhood in both sexes, and after puberty in males. As such, urogenital infant cytology should be useful in studies of early life exposure to endocrine disrupting chemicals. 

 While novel to the study of environmental endocrine disruption, estrogen-responsive epithelial cells have been well understood for decades and have been proposed as clinically informative biological endpoints in children. A predominance of superficial cells in the neonatal vaginal mucosa was noted as early as 1938 [23]; and, in the 1960s, superficial cells in urinary sediment were shown to decrease with increasing age in infancy and to rise with the onset of puberty in both males and females [12,13]. Clinically, a small study of children with endocrine-related disorders observed that 8 of 9 cases of premature thelarche (age 14-32 months) and 7 of 8 cases of gynecomastia (age 12-15 years) had ≥ 5% superficial cells in cells collected from urinary sediment, compared to 1 of 8 and 7 healthy control subjects, respectively [21]. 

Our study replicates the basic findings of several of these early studies and provides many practical considerations for study design, field application and sample analysis. In this respect, we observed that sample quality varied by sex, age at sampling, and collection method. Newborn males were prone to low cell counts and inflammation; urine specimens in this subpopulation were particularly problematic. These difficulties with newborn male sampling and specimen interpretation were possibly related to circumcision, but our data did not include circumcised state. In addition, newborn male specimen quality may have been compromised due to repeat sampling of the urinary meatus epithelial tissue: for example, if a swab was collected prior to a urine sample, the post-swab urethral tissue may have been depleted of informative cells at the time of urination. In a broader research context, however, it is important to note that newborns were sampled in this exercise only for the purpose of capturing a known period of high estrogen exposure. For future studies interested in elucidating responsiveness to exogenous estrogen exposures, this method would most suitably be applied to older infants, with low natural estrogen exposures. 

We also observed that two different scoring approaches yielded similar results. The NIEHS Pathologist tended to count more cells per specimen than the processing laboratory and applied more stringent inclusion criteria. As a result, the NIEHS Pathologist provided high quality readings on a limited number of specimens. Conversely, by using less stringent criteria, the processing laboratory’s results better represented the complete study sample. However, the laboratory’s results may have been more prone to cell-type misclassification, including false identification of cells as superficial due to ”pseudomaturation” (pyknotic nuclei), an effect of inflammation [9,11]. Such misclassification may have driven the unexpected difference in swab and urine specimens in newborn males in the laboratory analysis, as well as the overall tendency for the laboratory’s mean scores to be larger than the NIEHS Pathologist’s. Still, we reached the same overall conclusions from both approaches, so either approach can feasibly be taken in future studies. Sample size permitting, we expect that higher cell counts per specimen and stringent inclusion criteria will yield optimal results. 

## Conclusions

In this study, we have illustrated the potential for urogenital epithelial cells to be informative markers of estrogen response in healthy, term, normal birth weight infants. Additional study is needed to determine how epithelial cell characteristics might depend on gestational age, birth weight, and method of delivery, as well as demographic characteristics such as race. Ultimately, though, our findings support the extension of established cytological methods as a simple, inexpensive and minimally invasive way to detect a young child’s estrogenic response. We are currently conducting a study of infants fed soy formula who have relatively high exposure to the plant estrogens genistein and daidzein. We are using the method described here, as well as organ volume and concentrations of serum sex hormones, to establish the utility of different measures of estrogen response. As a result of this work, we hope that future studies will have methods, usable in infants, which credibly demonstrate biologically meaningful exposure to estrogens, including environmental endocrine disruptors. 
